# A floating elbow and an associated ipsilateral proximal humerus fracture: A case report

**DOI:** 10.1016/j.tcr.2025.101265

**Published:** 2025-11-14

**Authors:** Malick Diop, Mayoro Sow, Mohamed Daffé, Badara Dembele, Badara Diop, Khadim Seck, Andre Daniel Sane

**Affiliations:** a Centre Hospitalier National Dalal Jamm de Guediawaye, BP 19001, Dakar, Senegal; b Department of Surgery, Gaston Berger University, 234, Saint-Louis, National 2, route de Ngallèle, Saint-Louis, Senegal

**Keywords:** Floating elbow, Proximal humerus, Ipsilateral, Case report

## Abstract

We aim to describe this rare injury and evaluate the surgical treatment.

Floating elbow is a relatively rare traumatic entity. It occurs following a high-velocity trauma and is part of a polytrauma. There is no consensus on its management which remains essentially surgical. They often have a favorable outcome.

We report the case of a 20-year-old patient who presented following a road traffic accident with a floating elbow associated with an ipsilateral proximal humerus fracture. The treatment was surgical. An open reduction and internal fixation with a dual plating of the humerus and pinning of the forearm bones was carried out. At 10 months follow-up, the ASES score was 78. Rehabilitation was started early. The prognosis of this lesion is essentially functional.

## Introduction

Floating elbow is a relatively rare injury [1,2]. It occurs following a violent trauma. It is classically defined as a diaphyseal fracture of the humerus associated with an ipsilateral forearm fracture [3]. Recent definitions include articular fractures of the distal humerus [4,5].

The peculiarity of this case is the association of an ipsilateral proximal humerus fracture. This makes a floating elbow associated with a floating humeral shaft. This rare injury is part of a polytrauma. The patient was managed surgically. An open reduction and internal fixation with dual plating of the humerus, pinning of the forearm bones and intramedullary nailing of the tibia were performed under general anaesthesia. The outcome was favorable.

## Case report

Mr. ES, a 20-year-old right-handed patient was seen in the emergency room for polytrauma combining cervical spine injury, closed injury of the left upper limb and injury of the left leg. He was involved in a road traffic accident where he was allegedly hit by a bus while crossing the road.

Physical examination showed deformities of the left leg, forearm and arm, pain upon palpation of the cervical spinous processes with contraction of the cervical paravertebral muscles. The patient was non-neurological. There were no signs of radial nerve injury.

**Fig. 1:**
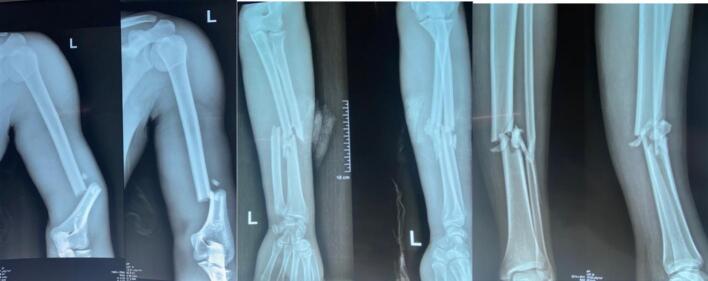
Plain X-rays of the arm, forearm and ipsilateral leg.

Radiological explorations showed: diaphyseal fractures of the left forearm bones associated with a segmental ipsilateral humerus fracture and fracture of both bones of the left leg (Fig. 1). There were no bone nor vertebro-medullary lesions of the cervical spine.

**Fig. 2:**
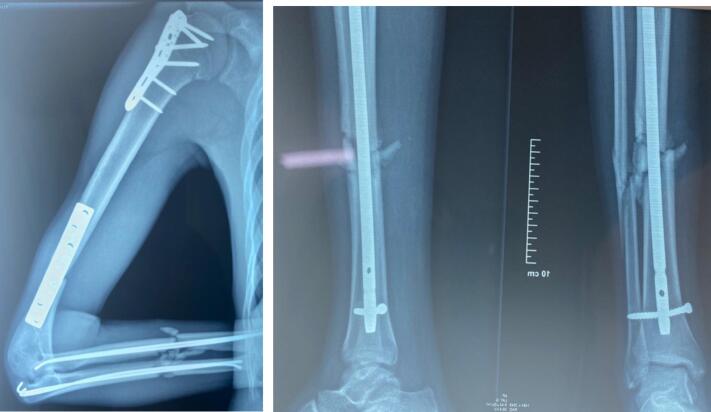
Post-operative X-rays.

The patient was managed in the operating room under general anaesthesia. A reduction and internal fixation with a locking proximal humerus plate and a Dynamic Compression Plate (DCP) was initially carried out for the humerus. Secondly, we performed a closed reduction and pinning of the ulna and radius under C-arm fluoroscopy. Finally, a closed reduction and intramedullary nailing of the left tibia was performed (Fig. 2). A cervical collar was placed for the spine.

The postoperative course was uneventful.

Rehabilitation was initiated and consisted of active and passive mobilization of the joints.

At 10 months follow-up, The ASES SCORE was 78/100. Activities of daily living were possible.

**Fig. 3:**
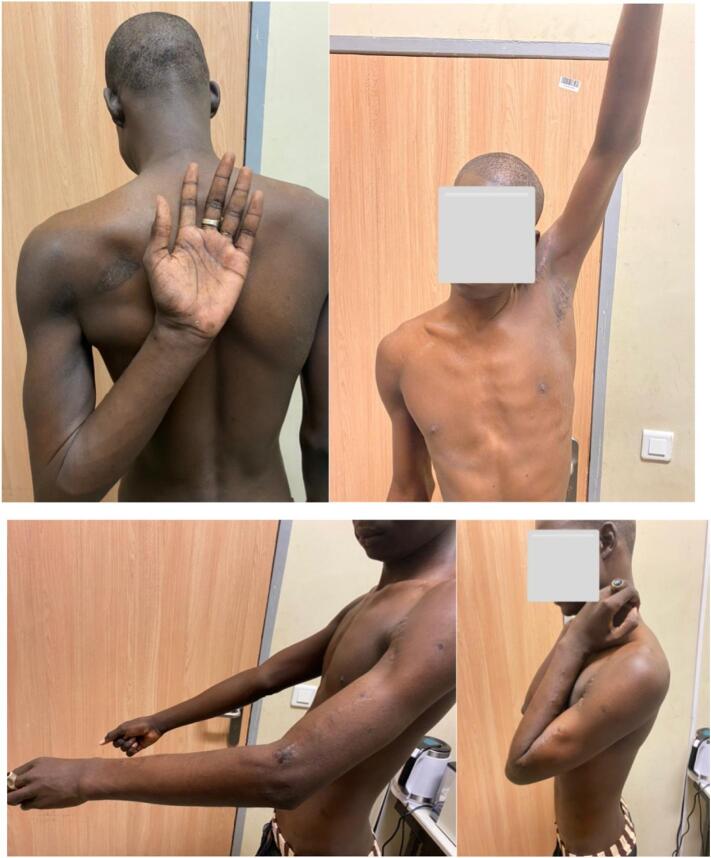
Mobilization joints at follow-up.

Shoulder antepulsion and abduction were 160 degrees. Rotations were complete. Elbow flexion was 120 degrees and extension was 45 degrees. Pronation-supination was limited (Fig. 3).

**Fig. 4:**
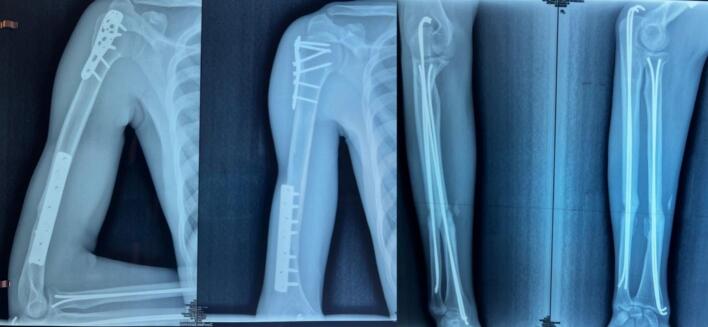
Radiographs of the arm (A) and forearm obtained at 12 months (M12) demonstrating fracture union.

At 12-month follow-up, radiographs demonstrated satisfactory fracture union (Fig. 4).

## Discussion

Floating elbow is a relatively rare injury. Most authors report isolated cases of floating elbow [1,2,6] or floating humerus [7,8]. Thus, the peculiarity of our case is the combination of a floating elbow and a floating humeral shaft. Such a combined lesion has not yet been found in the literature. It testifies to the violence of the trauma in our patient.

However, series with a larger number of cases exist in the literature [9,10]. It often occurs following high-velocity trauma and is sometimes part of a polytrauma. The mechanism of injury is unclear [2].

In this case report, the mechanism is probably multiple, combining a direct impact on the left side and a fall landing on the hand with an extended elbow. Constraints on the hyperpronated forearm could explain the radius and ulna fracture. The direct impact would have led to a staged fracture of the humerus.

The diagnosis is based on clinical and radiographic findings. In this context of polytrauma, whole-body computed tomography (WBCT) remains a very effective exploration to assess the various musculoskeletal, visceral and cerebral lesions among others. However, it should not delay treatment, especially in our regions.

Management should be undertaken as early as possible. Surgical treatment is universally accepted with various techniques ensuring stable fixation [1,11,12]. Several types of implants can be used. These include plates, external fixators and pins. Plating remains a very solid and stable fixation technique. We used a dual plate for the humerus and pinning for the forearm bones. Open reduction allowed us to obtain an anatomical reduction. There is no consensus on the surgical technique used. The best option for surgical treatment remains the most stable. In the context of polytrauma, damage control is the rule. However, complications may arise from this surgery. Surgical site infection and compartment syndrome are often described [1].

Rehabilitation is an essential step in treatment. It was started early in our patient. The short-term and intermediate-term outcomes were favorable in this patient with an ASES score of 78/100.

## Conclusion

A combined floating elbow and floating humerus is exceptional. It occurs in the context of violent trauma. A good strategy in diagnosis and management is necessary to obtain good outcomes. For this patient, the treatment was early, multidisciplinary and patient-specific.

## CRediT authorship contribution statement

**Malick Diop:** Writing – original draft, Conceptualization. **Mayoro Sow:** Visualization. **Mohamed Daffé:** Visualization. **Badara Dembele:** Visualization. **Badara Diop:** Visualization. **Khadim Seck:** Visualization. **Andre Daniel Sane:** Visualization, Validation, Supervision.

## Declaration of competing interest

The authors declare that they have no known competing financial interests or personal relationships that could have appeared to influence the work reported in this article.
